# The role of the C5a-C5aR pathway in iron metabolism and gastric cancer progression

**DOI:** 10.3389/fimmu.2024.1522181

**Published:** 2025-01-09

**Authors:** Qinxue Ni, Hong Yang, Hang Rao, Liyong Zhang, Mengyuan Xiong, Xiao Han, Boshao Deng, Lulu Wang, Jian Chen, Yan Shi

**Affiliations:** ^1^ The First Affiliated Hospital of Army Military Medical University, Department of General Surgery, Chongqing, China; ^2^ Department of Immunology, Army Medical University (Third Military Medical University), Chongqing, China

**Keywords:** C5a-C5aR pathway, gastric cancer, iron metabolism, macrophage polarization, LCN2, ER stress

## Abstract

Gastric cancer continues to be a leading global health concern, with current therapeutic approaches requiring significant improvement. While the disruption of iron metabolism in the advancement of gastric cancer has been well-documented, the underlying regulatory mechanisms remain largely unexplored. Additionally, the complement C5a-C5aR pathway has been identified as a crucial factor in gastric cancer development. The impact of the complement system on iron metabolism and its role in gastric cancer progression is an area warranting further investigation. Our research demonstrates that the C5a-C5aR pathway promotes gastric cancer progression by enhancing iron acquisition in tumor cells through two mechanisms. First, it drives macrophage polarization toward the M2 phenotype, which has a strong iron-release capability. Second, it increases the expression of LCN2, a high-affinity iron-binding protein critical for iron export from tumor-associated macrophages, by activating endoplasmic reticulum stress in these cells. Both mechanisms facilitate the transfer of iron from macrophages to cancer cells, thereby promoting tumor cell proliferation. This study aims to elucidate the connection between the complement C5a-C5aR pathway and iron metabolism within the tumor microenvironment. Our data suggest a pivotal role of the C5a-C5aR pathway in tumor iron management, indicating that targeting its regulatory mechanisms may pave the way for future iron-targeted therapeutic approaches in cancer treatment.

## 1 Introduction

Gastric cancer is a highly prevalent tumor that exhibits significant epidemiological variations in its incidence. Gastric cancer incidence is highest in East Asia and Eastern Europe and lowest in Africa. In 2022, there were more than 968,000 new cases of gastric cancer, leading to nearly 660,000 deaths, which positions it as the fifth most common cancer in terms of both incidence and mortality (1). Recent research indicates that the incidence rate of gastric cancer is rising among certain populations, particularly young individuals, potentially linked to dietary habits (2). Currently, the rate of early diagnosis for gastric cancer remains relatively low, especially in economically disadvantaged regions, where a significant number of patients are diagnosed at advanced stage (3). For those patients who have missed the surgical intervention opportunity, drug therapy has become the main treatment method. However, due to the adverse reactions and toxicity associated with drug therapy, such as nausea, vomiting, diarrhea, bone marrow suppression and so on, the overall treatment efficacy for gastric cancer remains inadequate, resulting in a low median overall survival rate (4). Relevant research indicates that the median survival time of advanced gastric cancer (GC) patients is less than one year, with a five-year survival rate of approximately 18% (5, 6). The prognosis for metastatic GC patients is particularly grim, as their median survival time is only 4 to 9 months (7). As treatment modalities continue to evolve, targeted therapy has emerged as a prominent area of interest, presenting new opportunities for gastric cancer management (8). Nevertheless, the current repertoire of targeted drugs for gastric cancer treatment is limited, underscoring the urgent need to identify novel therapeutic targets (9).

Iron participates in physiological activities such as cellular respiration, metabolism, DNA synthesis and repair in the human body, and is one of the essential trace elements for cell survival (10). Cancer cells exhibit an increased dependence on iron compared with normal cells. Iron metabolism disorders play a crucial role in tumor development, angiogenesis, invasion, and metastasis, presenting a common feature across various cancers. Research has demonstrated abnormal iron metabolism in lung cancer, prostate cancer, liver cancer, breast cancer, and kidney cancer (11–14). Additionally, some studies indicate that iron metabolism disorders are involved in the progression of gastric cancer. In gastric cancer, iron chelators induce gastric cancer cell apoptosis, involving endoplasmic reticulum stress formed by reactive oxygen species (ROS) and c-Jun N-terminal kinase activation (15). So, targeting iron metabolism may provide a new strategy for gastric cancer therapy.

To acquire sufficient iron necessary for cancer progression, cancer cells have developed various strategies to enhance iron uptake. Tumors can increase iron levels by upregulating the expression of iron import and storage proteins, such as transferrin receptor (TfR) and ferritin, while downregulating iron export proteins like ferroportin (FPN) (16). In the tumor microenvironment, macrophages can secrete lipocalin2 (LCN2) to elevate intracellular iron concentrations in tumor cells (17, 18). LCN2, also known as NGAL, plays a pivotal role in various physiological processes such as facilitating hydrophobic ligand transport across cell membranes, modulating immune responses, maintaining iron homeostasis, and promoting epithelial cell differentiation (19–21). In tumor progression, it can bind to iron-loaded siderophores, enabling tumor-infiltrating macrophages to release a continuous supply of iron to tumor cells (22). Importantly, LCN2 has been found to be abnormally expressed in a range of cancers, such as breast (23), colon (24, 25), and pancreatic (26), with recent studies highlighting its significant association with cancer initiation and progression. However, the function and role of LCN2 in gastric cancer (GC) remain enigmatic and a subject of debate.

Tumor associated macrophages (TAMs) are an important component of the tumor microenvironment. Compared with physiological inflammation, the TAM phenotype in tumors is more inclined towards the M2 phenotype (27). Research has shown that polarization of macrophages is closely related to iron metabolism, and M2 macrophages exhibit low ferritin and high iron transporter phenotypes, which are favorable for iron transport (28). As a regulator of iron within the organization and system homeostasis, these TAMs secrete iron and ferritin into the tumor matrix, thereby increasing tumor cell proliferation and metastasis. In addition to expressing transferrin receptors, TAMs also supply tumor cells with iron through the secreted LCN2. And previous research had proved that endoplasmic reticulum stress (ER stress) may also be involved in the role of macrophages in iron metabolism in the tumor microenvironment by upregulating LCN2 expression (29–31).

As macrophages play such a key role in cancer iron metabolism, so targeting macrophage polarization and iron-related genes regulation may provide new sight in cancer therapy. In tumor microenvironment, many immune cytokines are related to macrophage polarization or iron-related genes expression. Previous studies have shown that the expression of iron-related genes in macrophages is promoted by immune cytokines such as IL-6, IL-10 (32, 33). IL-4 could mediate M2 TAM polarization (34). Complement elements such as C3a or C5a appears to participate in some processes of the tumor progression, including the regulation of tumor angiogenesis and immune cells recruitment and phenotype (35, 36). Our previous research also provided evidence that breast cancer development may rely on C5a-C5aR interaction, for which MAPK/p38 pathway participated in downregulating the p21 expression. This suggests that the C5a-C5aR pathway plays an important role in the development of gastric cancer. Besides, it has been proved that C5a-C5aR pathway could lead to the polarization of TAMs toward M2 phenotype in ovarian cancer (37, 38). And Aiting Liu reported that C5a-C5aR pathway induced ER stress to accelerate vascular calcification (39). So, we speculate that complement C5/C5aR can regulate iron metabolism in gastric cancer through regulation macrophages polarization and LCN2 expression.

This study aims to explore the interaction between C5a-C5aR pathway and iron metabolism in gastric cancer, and it may provide new targets and ideas for the clinical treatment of gastric cancer.

## 2 Materials and methods

### 2.1 Patients and clinical specimens

Gastric cancer tissues and adjacent non-tumor tissues (5 cm from the tumor margin) were collected from 30 patients who underwent gastrectomy and lymph node dissection at Southwest Hospital (Chongqing, China) between 2021 and 2023. All patients had not been subjected to preoperative chemotherapy or radiotherapy. Ethical approval for the research was granted by the Ethics Committee of the First Affiliated Hospital of Army Medical University, PLA ((B)KY2023046). Participants were thoroughly informed about the sample processing and provided written consent prior to their inclusion in the study.

### 2.2 Bioinformatic analysis

Differential gene expression levels of LCN2, ER stress markers and C5aR1 between normal and gastric cancer (GC) tissues were analyzed using the Gene Expression Profiling Interactive Analysis 2 (GEPIA2) database (http://gepia2.cancer-pku.cn/#index), with a p-value threshold set at 0.01. GEPIA2 is an open-access online tool that enables interactive exploration of RNA sequencing data derived from 408 tumor samples and 211 normal samples within the TCGA-STAD cohort and the Genotype-Tissue Expression (GTEx) programs. The assessment of overall survival (OS) in patients correlated with varying levels of C5aR1 and LCN2 expression was performed using the online Kaplan-Meier plotter tool (https://kmplot.com/analysis/index.php?p=background).

### 2.3 Mouse model of GC

BALB/c female nude mice, sourced from Huafukang Co. (Beijing, China), were 6 weeks old at the experiment’s onset. They were randomly assigned to two groups and received subcutaneous transplantation of BGC-823 cells (~2×10^6^ BGC-823 cells into the subcutaneous on back). After tumor formation within three days, the mice were randomly assigned to two groups (n = 6 mice per group) and received intravenous injections every two days as follows: i) 0.9% NaCl, ii) C5aRA (1mg/kg) (GL Biochem, China), with tumor volume being monitored. The mice were sacrificed after 15 days, and the tumor tissues were promptly excised, weighed, and prepared for subsequent analysis via immunohistochemistry, immunofluorescence, flow cytometry and western blotting. Ethical approval for the animal research was granted by the Laboratory Animal Welfare and Ethics Committee of the Army Medical University (AMUWEC20242039).

### 2.4 Cell lines and cell culture

Human monocyte/macrophage (THP-1) cells purchased from the Procell Life Science &Technology (Wuhan, China) were cultured in Roswell Park Memorial Institute (RPMI) 1640 medium supplemented with 10% fetal bovine serum (Gibco, Life Technologies, USA) and maintained at 37°C in a humidified incubator with 5% CO_2_. RAW264.7 cells purchased from the Procell Life Science &Technology (Wuhan, China) were propagated in DMEM medium supplemented with 10% fetal bovine serum (Gibco, Life Technologies, USA) and maintained at 37°C in a humidified incubator with 5% CO_2_. To induce macrophage differentiation, THP-1 cells were cultured in 6-well plates, with 1×10^6^ cells per well, treated with 100 nM phorbol 12-myristate 13-acetate (PMA) (Sigma, Cat#P1585-1MG) for 24h. While RAW264.7 cells cultured in 6-well plates, with 5×10^5^ cells per well. Then, to elucidate the role of C5a, both cell types were stimulated with 100 ng/mL C5a (Beyotime Biotechnology, China) for 48 hours. In certain experiments, cells were pretreated with 10 nM C5aRA (GL Biochem, China) or 250 ng/mL ER stress antagonist, 4-Phenylbutyric acid (4-PBA) (Abmole, Cat#M9638-100mg, USA) or 10μM LCN2 inhibitor, ZINC00640089 (MedChemExpress, Cat#HY-Q45780, China) for 3 hours prior to C5a stimulation. After the 48-hour incubation period, cell samples were harvested for subsequent western blotting and quantitative RT-PCR analysis.

Gastric cancer cells including BGC-823 cells, HGC-27 cells, AGS cells were also cultured in RPMI 1640 medium supplemented with 10% fetal bovine serum (Gibco, Life Technologies, USA) and maintained at 37°C in a humidified incubator with 5% CO_2_. To ascertain the influence of C5a on gastric cancer cells via macrophages, we treated THP-1 cells as previously described. After a 6-hours incubation, the treatment medium was discarded and replaced with fresh 1640 medium. Subsequently, we collected distinct THP-1 macrophage culture supernatants and utilized them to cultivate gastric cancer cells for assessing cancer cells viability and iron content.

### 2.5 CCK-8 assay

Cell viability was determined using the CCK-8 assay (KEYGEN biotech, Jiangsu, China). Approximately 1×10^4^ gastric cells per well were seeded in 96-well plates with 100 μL medium each well. After a 6-hour incubation, the medium was replaced with PBS to serum-starve. Following an additional 12 hours, the PBS was substituted with different macrophage culture supernatants. After 24 hours of cultivation, each well was incubated with 10 μg CCK-8 for 2 hours in the dark. The absorbance was subsequently measured at 450 nm. Each treatment group was replicated 4 times for statistical analysis.

### 2.6 Iron content measurement

The intracellular iron content in cells was evaluated using Iron Content Assay Kit (Solarbio, China) following the manufacturer’s instructions, each group was replicated 3-5 times for statistical analysis.

### 2.7 Western blotting

GC or tumor-adjacent non-tumoral tissues and cell pellets were homogenized in Tissue Protein Extraction Reagent (Thermo Scientific, USA) with 1% protease inhibitor cocktail (CW Biotech, China). Then, equal amounts of total protein extracts from the cultured cells or tissues were separated using 8–12% SDS-PAGE and electro transferred onto the 0.45 μm PVDF membrane. Membranes were incubated with a monoclonal rabbit anti-human/mouse C5aR antibody (1:1000, Proteintech, Cat#21316-1-AP, China), LCN2 antibody (1:5000, Proteintech, Cat#26991-1-AP, China), GRP78 antibody (1:1000, Beyotime Biotechnology, Cat#AF0171, China), CHOP antibody (1:1000, Beyotime Biotechnology, Cat#AF6684, China), β-actin antibody (1:1000, Beyotime Biotechnology, Cat#AF5003, China), or α-tubulin antibody (1:1000, Beyotime Biotechnology, Cat#AF5012 China) and the respective HRP-conjugated secondary antibodies (1:1000; Beyotime Biotechnology, Cat#A0208, China) were used to detect the target proteins. The PVDF membrane was developed using ECL detection reagents (Advansta, USA) in a dark room. Results were normalized to the internal control β-actin or α-tubulin.

### 2.8 Histological, immunohistochemistry and multiplex immunofluorescence analysis

Human gastric tissues and mice tumor tissues were fixed in 4% (w/v) paraformaldehyde, routinely processed and embedded in paraffin and cut into 3-5 mm sections. Then some sections were stained with Perls blue stain kit (Solarbio, China) before microscopic evaluation at 200× or 400× magnification. For some sections, antigen retrieval was performed in citrate buffer (pH 6.0), endogenous peroxidase activity was quenched with 3% H_2_O_2_ solution, and nonspecific binding was blocked with bovine serum albumin. For immunohistochemistry staining, the expression of C5aR, LCN2, GRP78 and CHOP in mice tumor tissues and human gastric tissues was detected with a monoclonal rabbit anti-human/mouse C5aR antibody (1:200, Proteintech, Cat#21316-1-AP), LCN2 antibody (1:200, Proteintech, Cat#26991-1-AP), GRP78 antibody (Beyotime Biotechnology, Cat#AF0171), CHOP antibody (1:200, Beyotime Biotechnology, Cat#AF6684),. After overnight in the refrigerator at 4℃, secondary antibody incubation was carried out with HRP rabbit antibody (Beyotime Biotechnology, Cat#A0208). DAB kit was used for visualization, and nuclei stained with hematoxylin. For multiplex immunofluorescence, the expression of CD206 in mice tumor tissues was detected with a monoclonal rabbit anti-mouse CD206 antibody (1:200, Proteintech, Cat#18704-1-AP). Secondary antibody incubation was carried out with HRP rabbit antibody, after overnight in the refrigerator at 4℃. Visualization of CD206 was accomplished using Rhod B TSA Plus (1:100), after which the sections were placed in citrate buffer and heated again. Then the expression of iNOS (1:200, Proteintech, Cat#18985-1-AP) in mice tumor tissues was detected in same way while visualization of iNOS was accomplished using Alexa 488 TSA Plus. Nuclei were subsequently visualized with DAPI (1:2000).

### 2.9 Quantitative RT-PCR

Total RNA was isolated from cultured cells using the RNA easy™ Animal RNA Isolation Kit with Spin Column (R0026; Beyotime Biotechnology, Shanghai, China). We followed the manufacturer’s instructions to reverse transcribe RNA using the Prime Script™ RT kit and gDNA Eraser (Perfect Real Time) (RR047A, Takara, Japan). Quantitative RT-PCR was used to quantify the target cDNA levels, and the Bio-Rad CFX96 detection system along with TB Green^®^ Premix Ex Taq™ II (RR820A, Takara, Japan) was employed to assess mRNA levels. The relative expression levels of each gene were normalized to the β-actin gene. And each group was replicated 3 times for statistical analysis. The following primer sequences were used: Human β-actin: Forward 5’-3’ CACTCTTCCAGCCTTCCTTC, Reverse 5’-3’ GTACAGGTCTTTGCGGATGT; human CCR7: Forward 5’-3’TGGTGGTGGCTCTCCTTGTC, Reverse5’-3’TGTGGTGTTGTCTCCGATGTAATC; human CD206: Forward 5’-3’ TCCGGGTGCTGTTCTCCTA, Reverse 5’-3’ CCAGTCTGTTTTTGATGGCACT; human LCN2: Forward 5’-3’, TCACCTCCGTCCTGTTTAGG; Reverse 5’-3’, CGAAGTCAGCTCCTTGGTTC; human ATF4: Forward 5’-3’ ATGACCGAAATGAGCTTCCTG, Reverse 5’-3’ GCTGGAGAACCCATGAGGT; human CHOP: Forward 5’-3’ GGAAACAGAGTGGTCATTCCC, Reverse 5’-3’ CTGCTTGAGCCGTTCATTCTC; human GRP78: Forward 5’-3’ GCCTGTATTTCTAGACCTGCC, Reverse 5’-3’ TTCATCTTGCCAGCCAGTTG; mouse β-actin: Forward 5’-3’ GGCTCTTTTCCAGCCTTCCT, Reverse 5’-3’ GTCTTTACGGATGTCAACGTCACA; mouse CD206: Forward 5’-3’ CTCTGTTCAGCTATTGGACGC, Reverse 5’-3’ TGGCACTCCCAAACATAATTTGA; mouse CCL3: Forward 5’-3’ CATATGGAGCTGACACCCCG, Reverse 5’-3’ GAGCAAAGGCTGCTGGTTTC; mouse CHOP: Forward 5’-3’ AAGCCTGGTATGAGGATCTGC; Reverse 5’-3’ TTCCTGGGGATGAGATATAGGTG; mouse ATF4: Forward 5’-3’ CCTGAACAGCGAAGTGTTGG, Reverse 5’-3’ TGGAGAACCCATGAGGTTTCAA; mouse GRP78: Forward 5’-3’ ACTTGGGGACCACCTATTCCT, Reverse 5’-3’ GTTGCCCTGATCGTTGGCTA; mouse LCN2: Forward 5’-3’ TGGCCCTGAGTGTCATGTG, Reverse 5’-3’ CTCTTGTAGCTCATAGATGGTGC.

### 2.10 Flow cytometry

The nude mice tumor tissues were digested with Tumor Dissociation Kit (Miltenyi Biotec, Cat#130-096-730) using gentleMACSTM Octo Dissociator with Heaters (Miltenyi Biotec) and then crushed through mesh for single cell suspension. To determine macrophage polarization, cells from tumor tissues were stained with Zombie. NIRTM Dye (BioLegend, Cat#B323327), anti-mouse CD45 (BioLegend, Cat#103132), CD11b (BioLegend, Cat#101263), F4/80 (BioLegend, Cat#123110), I-A/I-E (BioLegend, Cat#107605), and CD206 (BioLegend, Cat#141717). After staining process by following the manufacturer’s instructions, samples were analyzed by flow cytometry (CytoFLEXTM).

### 2.11 Statistical analysis

Statistical analyses were executed using GraphPad Prism 9 software, with comparisons made via unpaired two-tailed Student’s t-test or one-way ANOVA as appropriate. Data are presented as mean ± standard deviation (SD). Significance levels were set at p<0.05 (denoted by *), p<0.01 (denoted by **), p<0.001 (denoted by ***), and p<0.0001 (denoted by ****).

## 3 Results

### 3.1 Gastric cancer displayed iron accumulation, LCN2 up-regulation and ER stress activation

**Figure 1:**
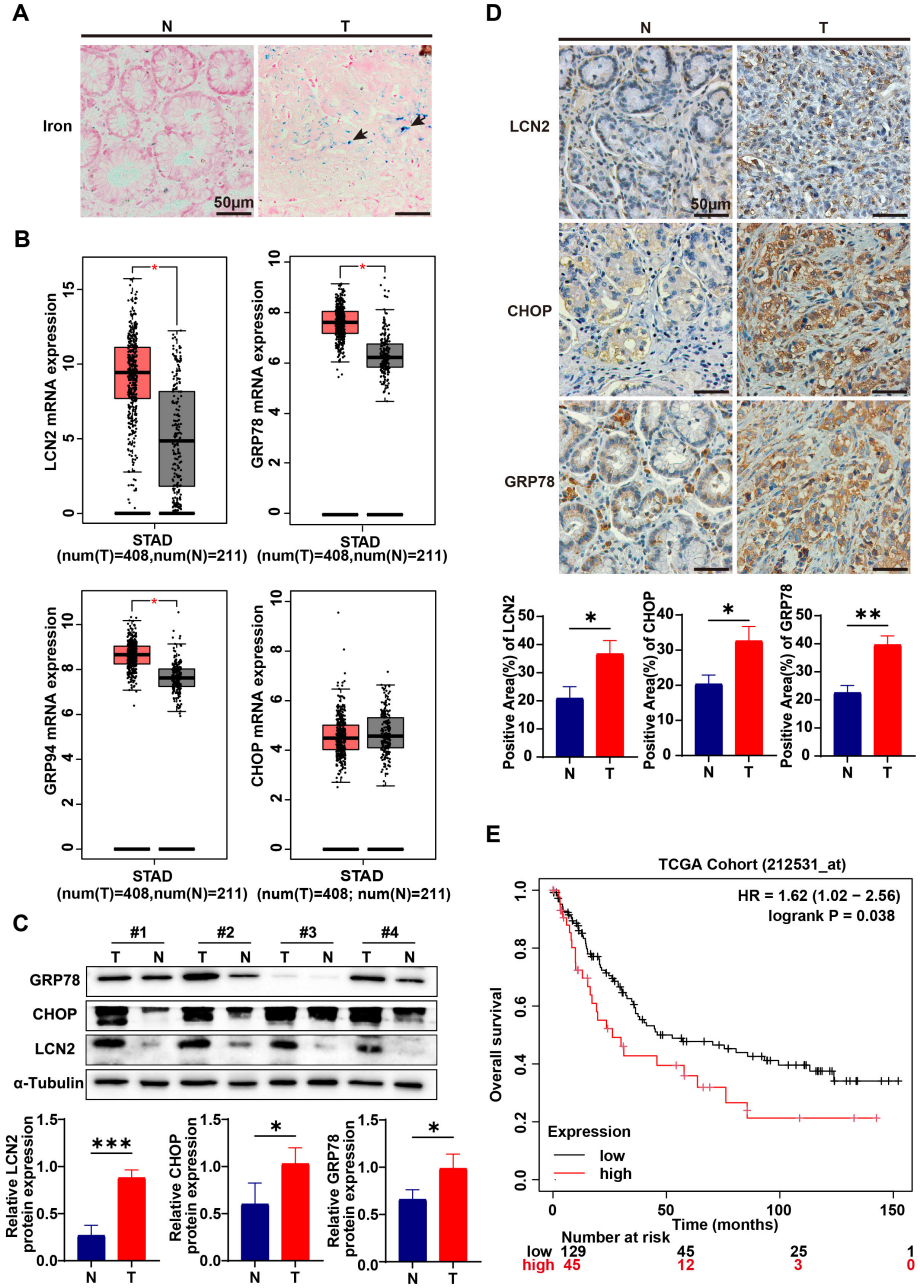
Iron accumulation and LCN2, ER stress up-regulation in gastric cancer. **(A)** By Perls’ blue staining on gastric cancer tissue (T) and adjacent normal tissue (N) from clinical patients, the accumulation of iron in gastric cancer tissue and paired adjacent normal tissue was detected. The blue color indicated by the arrow in the figure represent iron deposited in the tissue (representative panels scale bar, 50 μm). **(B)** Quantification of lipocalin2 (LCN2) and endoplasmic reticulum stress (ER stress) marker genes, such as Glucose Regulated Protein 78, 94 (GRP78, GRP94) and CCAAT/Enhancer-Binding Protein Homologous Protein (CHOP) mRNA expression in gastric cancer tissues and normal gastric tissues in the STAD cohort and GTEx. Data were analyzed using the Gene Expression Profiling Interactive Analysis 2 (GEPIA2) database (http://gepia2.cancer-pku.cn/#index), with a p-value threshold set at 0.01. **(C)** Expression levels of LCN2, CHOP and GRP78 in GC and paired adjacent normal tissues were examined using western blot analysis, results were normalized to the internal control α-tubulin, and the relative protein expression levels were statistically analyzed (n=3-4). **(D)** LCN2, CHOP and GRP78 expression in GC and paired adjacent normal tissues were determined by IHC (representative panels scale bar, 50 μm), and statistically analyzed the percentage of positive areas (n=3). **(E)** The assessment of overall survival (OS) in patients correlated with varying levels of LCN2 expression was performed using the online Kaplan-Meier plotter tool (https://kmplot.com/analysis/index.php?p=background). Statistical analyses were executed using GraphPad Prism 9 software, with comparisons made via unpaired two-tailed Student’s t-test. Data are presented as mean ± standard deviation (SD). Significance levels were set at p<0.05 (denoted by *), p<0.01 (denoted by **), p<0.001 (denoted by ***).

Studies have demonstrated that cancer cells exhibit a greater demand for iron to sustain their proliferative capacity, with dysregulated iron metabolism recognized as one of the metabolic hallmarks of malignant cells (40). To investigate the role of iron in gastric cancer progression, Perls’ blue staining was utilized to assess iron accumulation in both gastric cancer (GC) and normal tissues. Results indicated that GC tissues exhibited significantly higher iron accumulation compared to their normal counterparts (**Figure 1A**). This suggests that gastric cancer likely develops strategies to enhance iron uptake and intracellular storage to support its elevated metabolic demands. Several genes, including the transferrin receptor, ferritin heavy chain, ferritin light chain, iron exporter ferroportin, iron regulatory protein 2, and LCN2, have been implicated in the regulation of iron in cancer cells. Notably, LCN2 expression in tumors has been associated with poor prognosis and is believed to facilitate tumor development and metastatic spread in endometrial and breast cancer (41, 42). However, the effect of LCN2 on iron metabolism in gastric cancer has not been investigated to date. To ascertain whether LCN2 is associated with iron accumulation in gastric cancer, we first analyzed the cohort data from TCGA. The results indicated that LCN2 expression in gastric cancer tissues was significantly higher than in normal gastric tissues (**Figure 1B**). Subsequently, we conducted Western blot and immunohistochemical staining on clinical specimens, confirming that the protein levels of LCN2 in gastric cancer tissues were elevated compared to adjacent tissues (**Figures 1C, D**). Therefore, gastric cancer may upregulate LCN2 to meet its iron demands. Additionally, Navin R Mahadevan reported that ER stress drives LCN2 upregulation in prostate cancer cells (43). This leads us to suspect that LCN2 upregulation in gastric cancer could also be linked to ER stress. To investigate this, we analyzed the TCGA cohort data, revealing that the expression levels of ER stress markers, such as Glucose Regulated Protein 78 and 94 (GRP78 and GRP94), were significantly higher in gastric cancer tissues than in normal gastric tissues (**Figure 1B**). Results from Western blot and immunohistochemical staining of clinical specimens further demonstrated elevated protein levels of ER stress markers, including CCAAT/Enhancer-Binding Protein Homologous Protein (CHOP) and GRP78, in gastric cancer tissues compared to adjacent tissues (**Figures 1C, D**). Furthermore, survival analysis using data from the KMPLOT website indicated a correlation between LCN2 expression levels and patient prognosis in gastric cancer. Patients with lower LCN2 expression showed an overall improvement in survival rate (**Figure 1E**). We proposed that upregulation of ER stress and LCN2 may facilitate iron transport to gastric cancer cells, thus accelerating its advancement. Given that LCN2 may play a crucial role in gastric cancer iron metabolism, exploring the mechanisms regulating LCN2 could provide a theoretical foundation for developing it as a novel therapeutic target for gastric cancer.

### 3.2 C5a-C5aR pathway activation in gastric cancer

Accumulating evidence suggests that the complement system plays a crucial role in regulating cancer progression, thus contributing, to varying degrees, to tumor initiation and development (36, 44). It has the potential to reprogram tumor-associated macrophages (TAMs) and promote their polarization toward the M2 phenotype. Additionally, it can induce endoplasmic reticulum stress, thereby accelerating vascular calcification (39). However, there has not yet to be a study investigating the correlation between the complement system and iron metabolism in gastric cancer. Given that LCN2 is secreted by M2 phenotype TAMs and regulated by ER stress, we hypothesize that the C5a-C5aR pathway may enhance macrophage polarization toward the M2 phenotype and activate ER stress, ultimately increasing LCN2 secretion. This process could provide more iron to gastric cancer, thereby accelerating its progression.

**Figure 2:**
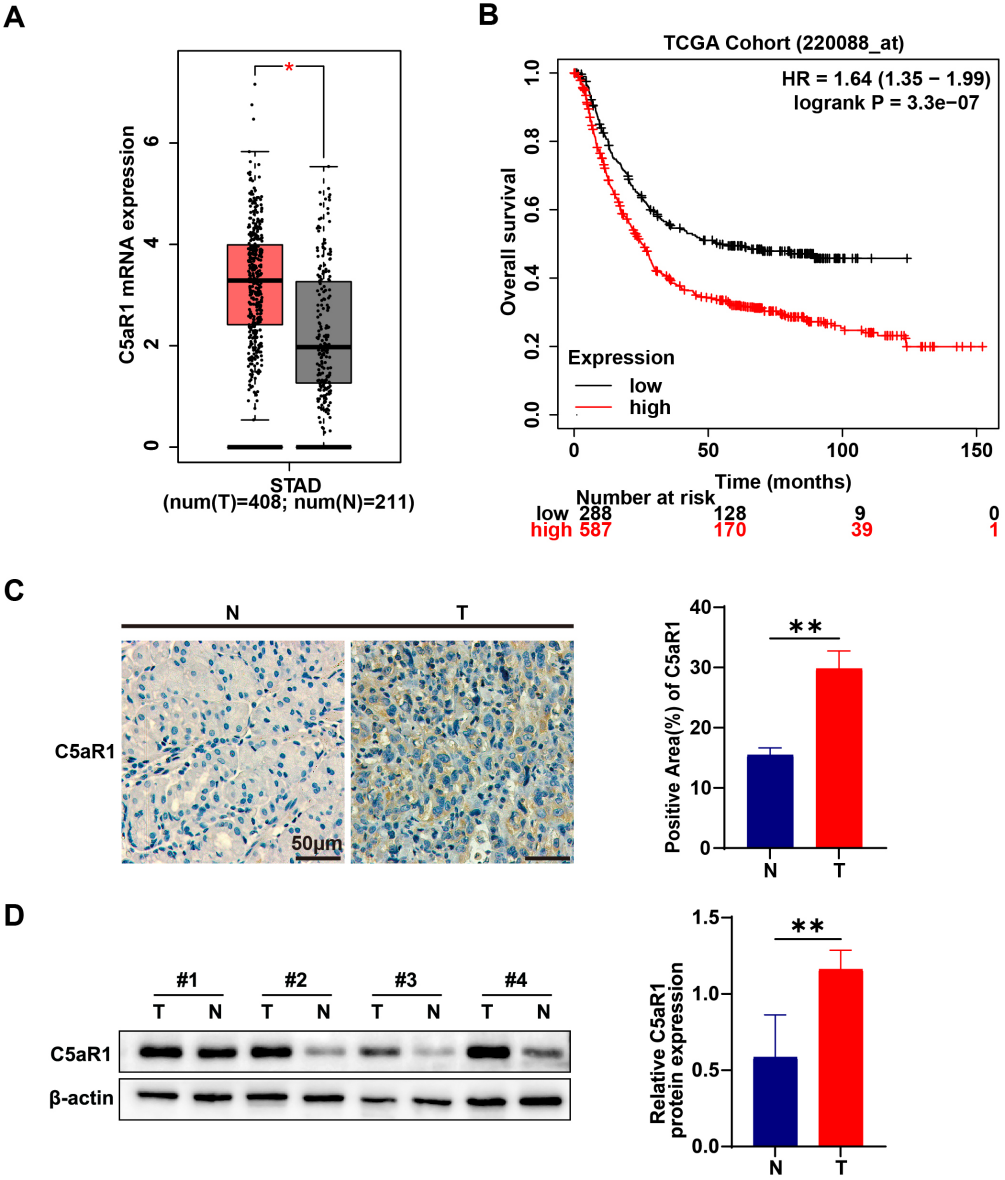
C5a-C5aR pathway activation in gastric cancer. **(A)** Quantification of C5AR1 mRNA expression in tumor tissues and normal gastric tissues in the STAD cohort and GTEx. Data were analyzed using the GEPIA2 database. **(B)** The assessment of overall survival (OS) in patients correlated with varying levels of C5aR1 expression was performed using the online Kaplan-Meier plotter tool. **(C)** C5aR1 expression in GC and paired adjacent normal tissues were determined by IHC (representative panels scale bar, 50 μm), and statistically analyzed the percentage of positive areas (n=3). **(D)** Protein levels of C5aR1 in GC and paired adjacent normal tissues were examined using western blot analysis results were normalized to the internal control β-actin, and the relative protein expression levels were statistically analyzed (n=4). Statistical analyses were executed using GraphPad Prism 9 software, with comparisons made via unpaired two-tailed Student’s t-test. Data are presented as mean ± standard deviation (SD). Significance levels were set at p<0.05 (denoted by *), p<0.01 (denoted by **).

We explored alterations in C5aR1 expression within gastric cancer (GC). Analysis of C5aR1 mRNA levels in gastric tumor tissues, sourced from the bioinformatics TCGA-STAD cohort and the Genotype-Tissue Expression (GTEx) project, revealed a significant upregulation compared to normal gastric tissues (**Figure 2A**). Survival analysis using data from the KMPLOT website indicated a correlation between C5aR1 expression levels and patient prognosis in gastric cancer, with patients exhibiting lower C5aR1 expression experiencing improved overall survival rates (**Figure 2B**). Furthermore, we examined C5aR1 expression in paired GC and normal tissues from clinical specimens, confirming that C5aR1 levels were elevated in GC tissues relative to adjacent normal tissues (**Figures 2C, D**). Collectively, these findings underscore the activation of the C5a-C5aR pathway in gastric cancer.

### 3.3 C5a-C5aR pathway promoted gastric cancer progression by increasing iron transfer from macrophages to cancer cells

**Figure 3:**
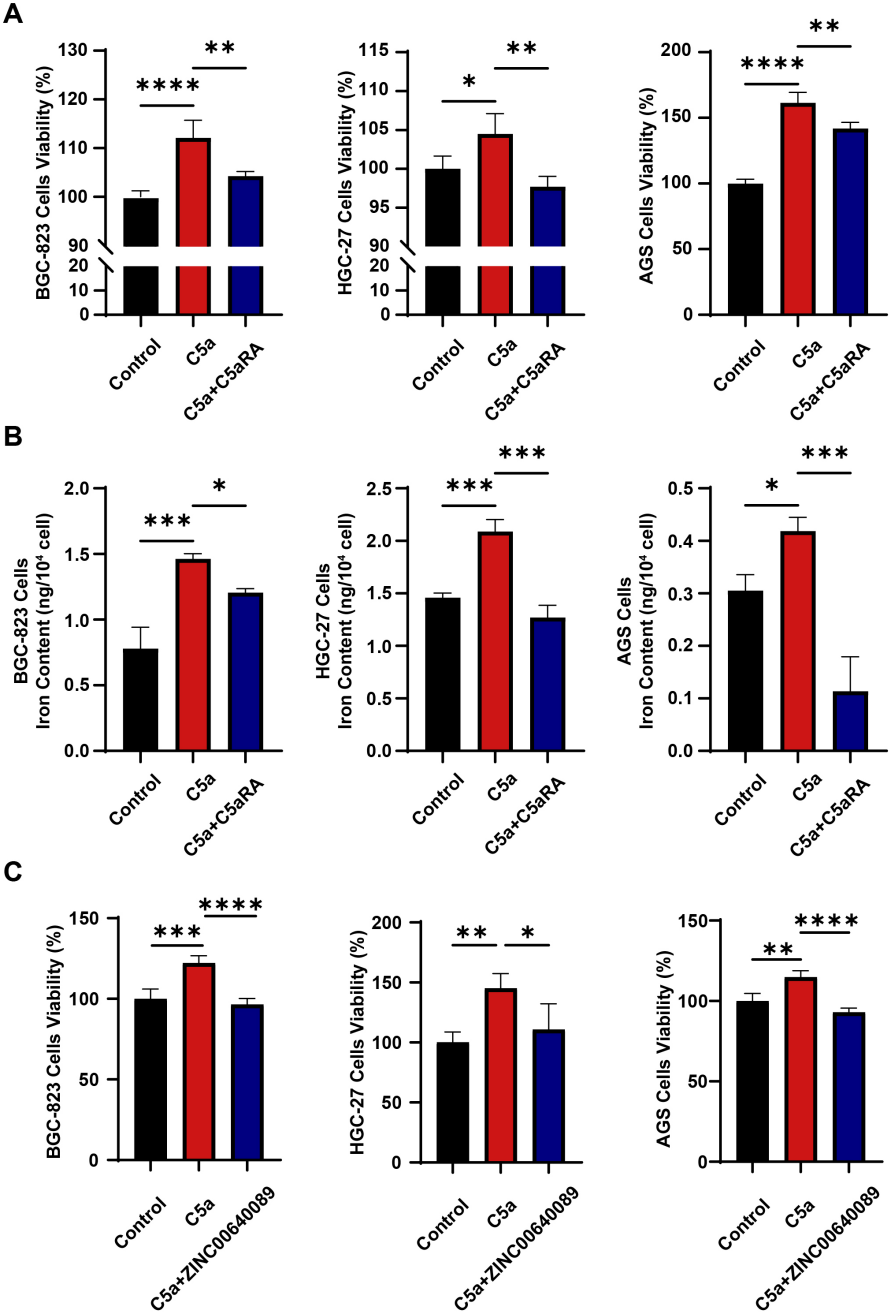
C5a-C5aR pathway enhanced gastric cancer progression by facilitating iron transfer from macrophages to cancer cells. THP-1 cells were cultured in 6-well plates, with 1×10^6^ cells per well, pre-treated with 100 nM phorbol 12-myristate 13-acetate (PMA) for 24h. Then, in certain experiments, cells were pretreated with 10 nM C5aR antagonist (C5aRA) or 10μM LCN2 inhibitor, ZINC00640089 for 3 hours, followed by stimulation with 100 ng/mL recombinant human C5a protein. After a 6-hours incubation, the treatment medium was discarded and replaced with fresh 1640 medium. Subsequently, distinct THP-1 macrophage culture supernatants were collected and utilized to cultivate gastric cancer cells for assessing cancer cells viability or iron content. **(A)** Cell viability of gastric cancer cells including BGC-823 cells, HGC-27 cells, AGS cells culturing in different culture medium (Control, C5a or C5a+C5aRA) was examined using CCK8 assay (n=4). **(B)** Intracellular iron content in gastric cancer cells was tested by Iron Content Assay Kit (n=3~5). **(C)** Cell viability of gastric cancer cells including BGC-823 cells, HGC-27 cells, AGS cells culturing in different culture medium (Control, C5a or C5a+ZINC00640089) was examined using CCK8 assay (n=4). Statistical analyses were executed using GraphPad Prism 9 software, with comparisons made via one-way ANOVA test. Data are presented as mean ± standard deviation (SD). Significance levels were set at p<0.05 (denoted by *), p<0.01 (denoted by **), p<0.001 (denoted by ***), and p<0.0001 (denoted by ****).

To verify the role of C5a-C5aR pathway in gastric cancer progression, we cultured three types of gastric cancer cell lines using THP-1 macrophage supernatant treated under various conditions as the culture medium. As illustrated in **Figure 3A**, the supernatant from macrophages stimulated by C5a promotes the proliferation of gastric cancer cells; however, this effect is partially diminished by the addition of C5aRA. Subsequently, we measured the intracellular iron content of the gastric cancer cells. As depicted in **Figure 3B**, supernatants of macrophages treated with C5a enhanced the iron content in all three types of gastric cancer cells, and C5aRA weakened this effect. Our research findings indicated that C5a-C5aR pathway promoted gastric cancer progression by enhancing iron transporting from macrophages to gastric cancer cells. To further investigate whether C5a/C5aR pathway-induced proliferation of gastric cancer cells occurs via LCN2, we treated THP1 cells with C5a and C5a+ ZINC00640089 (an LCN2-specific inhibitor). The supernatant was subsequently collected to culture gastric cancer cells. The results in **Figure 3C** indicated that the C5a/C5aR pathway could promote gastric cancer progression, however, this effect was inhibited by the LCN2 inhibitor. These findings collectively suggested that the C5a/C5aR pathway stimulated gastric cancer cell proliferation through LCN2, highlighting the critical role of LCN2 in gastric cancer progression.

### 3.4 C5a-C5aR pathway enhanced the polarization of macrophages towards the M2 phenotype *in vitro*

**Figure 4:**
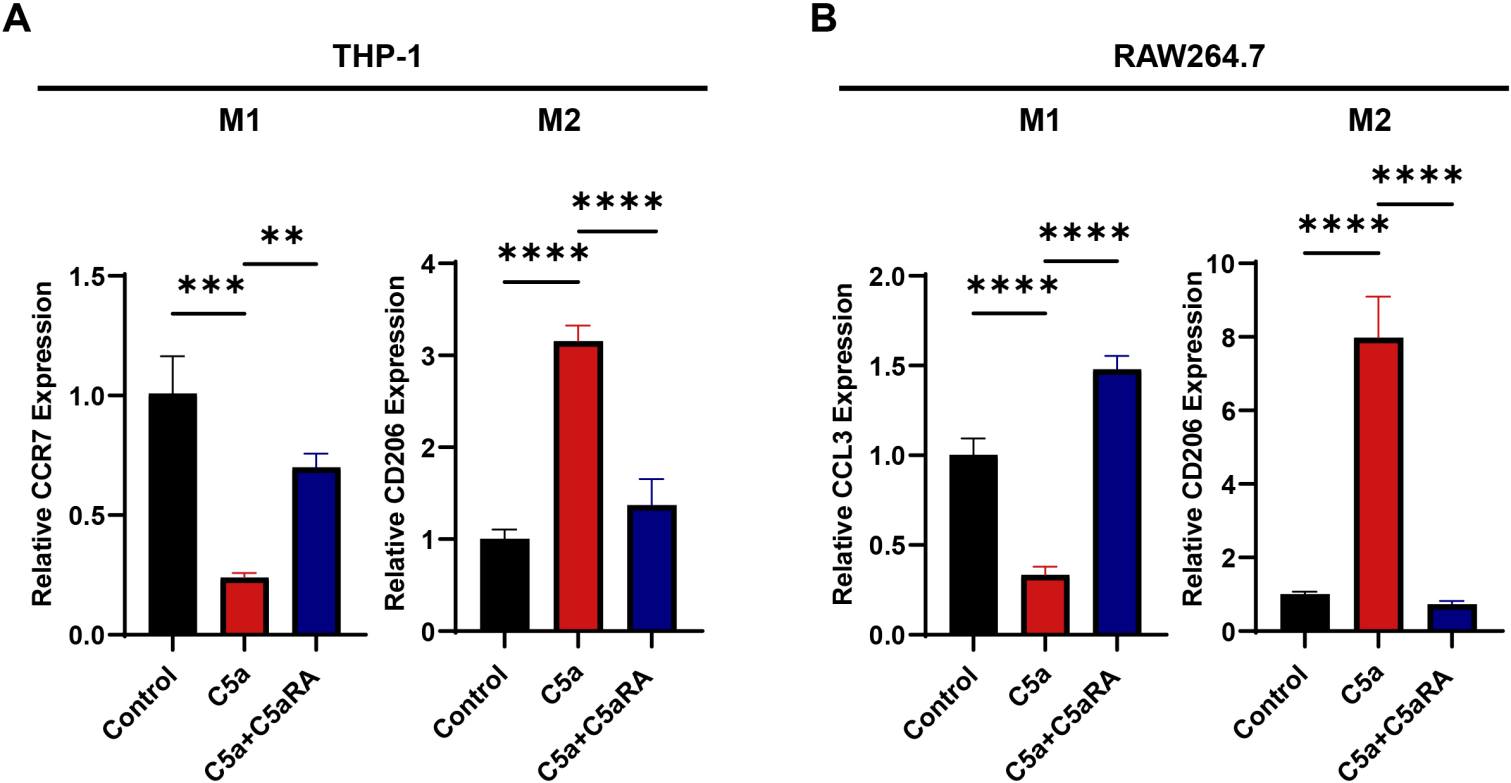
C5a-C5aR pathway promoted macrophages polarization to M2 phenotype *in vitro*. THP-1 cells were cultured in 6-well plates, with 1×10^6^ cells per well, pre-treated with 100 nM phorbol 12-myristate 13-acetate (PMA) for 24h. While RAW264.7 cells were cultured in 6-well plates, with 5×10^5^ cells per well. Then both cell types were stimulated with 100 ng/mL C5a for 48 hours. In certain experiments, cells were pretreated with 10 nM C5aRA for 3 hours prior to C5a stimulation. After the 48-hour incubation period, cell samples were harvested for quantitative RT-PCR analysis. **(A)** Relative gene expression of CCR7 and CD206 in THP-1 cell treated with different group (Control, C5a or C5a+C5aRA) were examined by QRT-PCR (n=3). **(B)** Relative gene expression of CCL3 and CD206 in RAW264.7 cell treated with different group (Control, C5a or C5a+C5aRA) were examined by QRT-PCR (n=3). Statistical analyses were executed with one-way ANOVA test. Data are presented as mean ± standard deviation (SD). Significance levels were set at p<0.01 (denoted by **), p<0.001 (denoted by ***), and p<0.0001 (denoted by ****).

Previous studies have demonstrated that M2 phenotype macrophages exhibit increased phagocytic activity, efficiently recycle iron, produce and secrete the iron transporter LCN2 into their microenvironment, thereby supplying more iron to cancer cells (17). To further investigate the specific mechanism by which the C5a-C5aR pathway regulates iron metabolism, we first examined its effect on macrophage polarization. We conducted *in vitro* stimulation experiments using THP-1 and RAW264.7 cells. THP-1 cells were seeded in 6-well plates at a density of 1 × 10^6^ cells per well and treated with PMA for 24 hours, while RAW264.7 cells were seeded at a density of 5 × 10^5^ cells per well. Following this, both cell types were treated with recombinant C5a protein at a concentration of 100 ng/mL for 48 hours, with some assays involving a pretreatment with 10 nM C5aRA. RNA was extracted for QRT-PCR analysis. After C5a treatment, the expression levels of M1 macrophage marker genes CCR7 and CCL3 decreased, while the expression of the M2 polarization marker gene CD206 increased (**Figures 4A, B**). These results confirmed that C5a-C5aR pathway promoted macrophages polarization to M2 phenotype *in vitro*.

### 3.5 C5a-C5aR pathway stimulated the expression of LCN2 through the induction of endoplasmic reticulum stress

**Figure 5:**
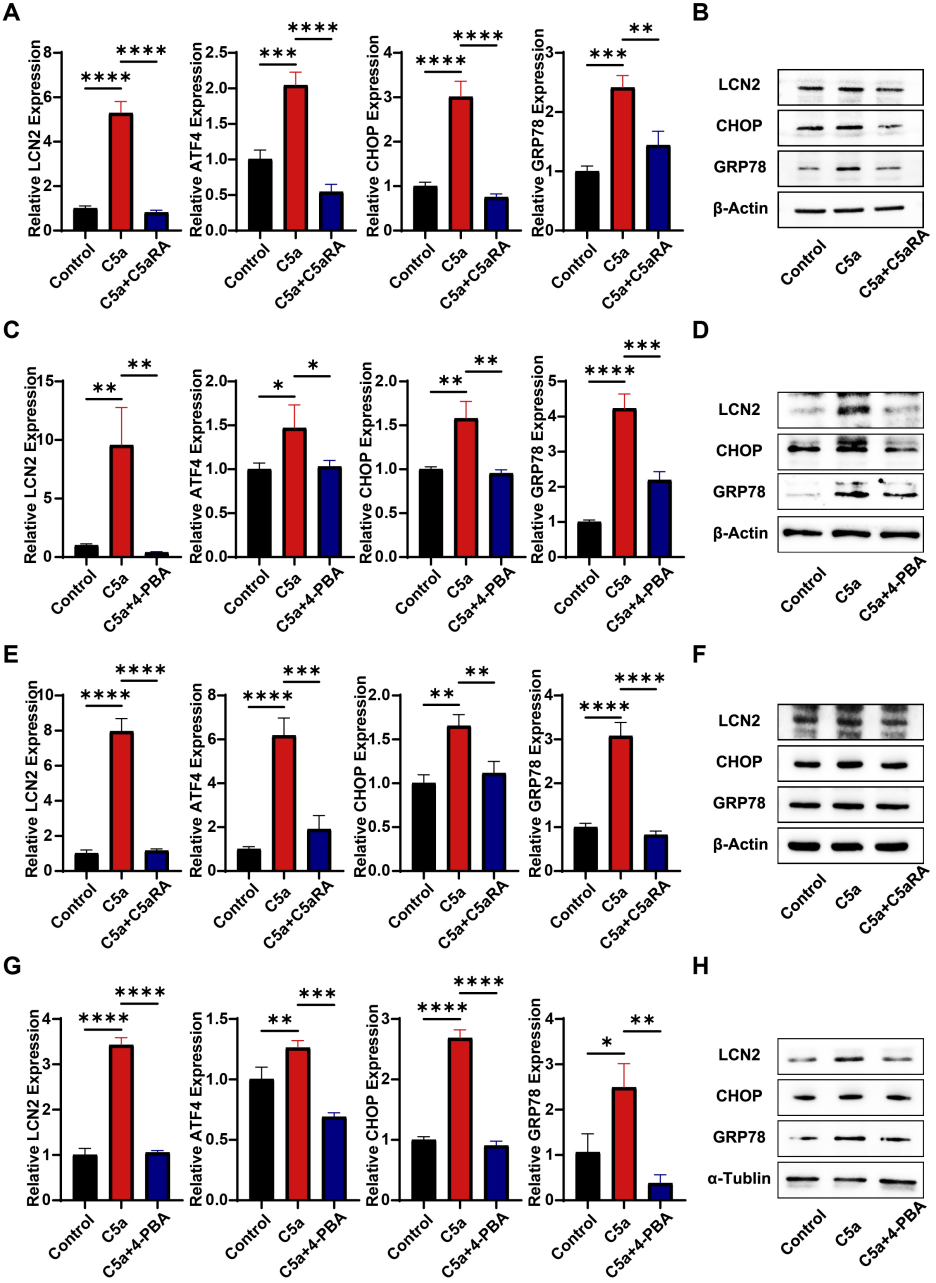
C5a-C5aR promoted LCN2 expression via activation of ER stress in macrophages. PMA pre-treated THP-1 cells and RAW264.7 cells were stimulated with 100 ng/mL C5a for 48 hours. In certain experiments, cells were pretreated with 10 nM C5aRA or 250 ng/mL ER stress antagonist, 4-Phenylbutyric acid (4-PBA). After the 48-hour incubation period, cell samples were harvested for subsequent western blotting and quantitative RT-PCR analysis to detect LCN2 and ER stress related markers (CHOP, GRP78 and activating transcription factor 4, ATF4). **(A)** Relative gene expression of LCN2, ATF4, CHOP and GRP78 in THP-1 cell treated with different group (Control, C5a or C5a+C5aRA) were examined by QRT-PCR (n=3). **(B)** Protein levels of GRP78, CHOP and LCN2 in THP-1 cell treated with different group (Control, C5a or C5a+C5aRA) were examined using western blot analysis. **(C)** Relative gene expression of ATF4, CHOP, GRP78 and LCN2 in THP-1 cell treated with different group (Control, C5a or C5a+4-PBA) were examined by QRT-PCR (n=3). **(D)** Protein levels of GRP78, CHOP and LCN2 in THP-1 cell (Control, C5a or C5a+4-PBA) were examined using western blot analysis. **(E)** Relative gene expression of ATF4, CHOP, GRP78 and LCN2 in RAW264.7 cell treated with different group (Control, C5a or C5a+C5aRA) were examined by QRT-PCR (n=3). **(F)** Protein levels of GRP78, CHOP and LCN2 in RAW264.7 cell treated with different group (Control, C5a or C5a+C5aRA) were examined using western blot analysis. **(G)** Relative gene expression of ATF4, CHOP, GRP78 and LCN2 in RAW264.7 cell treated with different group (Control, C5a or C5a+4-PBA) were examined by QRT-PCR (n=3). **(H)** Protein levels of GRP78, CHOP and LCN2 in RAW264.7 cell treated with different group (Control, C5a or C5a+4-PBA) were examined using western blot analysis. Statistical analyses were executed with one-way ANOVA test. Data are presented as mean ± standard deviation (SD). Significance levels were set at p<0.05 (denoted by *), p<0.01 (denoted by **), p<0.001 (denoted by ***), and p<0.0001 (denoted by ****).

Besides its effects on macrophage polarization, we hypothesize that the C5a-C5aR pathway may upregulate LCN2 by activating endoplasmic reticulum (ER) stress, thereby enhancing iron transport to cancer cells. This assumption is supported by a previous study by Aiting Liu, which reported that the C5a-C5aR pathway induced ER stress to accelerate vascular calcification (39). To investigate the impact of the C5a-C5aR pathway on LCN2 and ER stress, THP-1 cells and RAW264.7 cells were pretreated with 10 nM C5aRA for 3 hours, followed by treatment with 100 ng/mL recombinant C5a protein for 48 hours. QRT-PCR and western blot analyses were conducted. As shown in **Figures 5A, B**, both mRNA and protein levels of LCN2, along with ER stress-related markers in THP-1 cells, were significantly elevated in the C5a-treated groups. These effects were partially inhibited by the addition of the C5aRA, suggesting that the C5a-C5aR pathway promoted LCN2 expression and activated ER stress in macrophages. To further validate that C5a-C5aR regulates LCN2 by activating ER stress, cells were pretreated with the ER stress antagonist 4-PBA before stimulation with recombinant C5a. QRT-PCR and western blot assays demonstrated that 4-PBA effectively reversed the C5a-induced upregulation of LCN2 (**Figures 5C, D**), indicating that the C5a-C5aR pathway enhanced LCN2 expression through ER stress activation. Notably, the experimental results in RAW264.7 cells were consistent with those observed in THP-1 cells (**Figures 5E–H**).

### 3.6 Inhibition of the C5a-C5aR pathway suppressed the progression of gastric cancer *in vivo*

**Figure 6:**
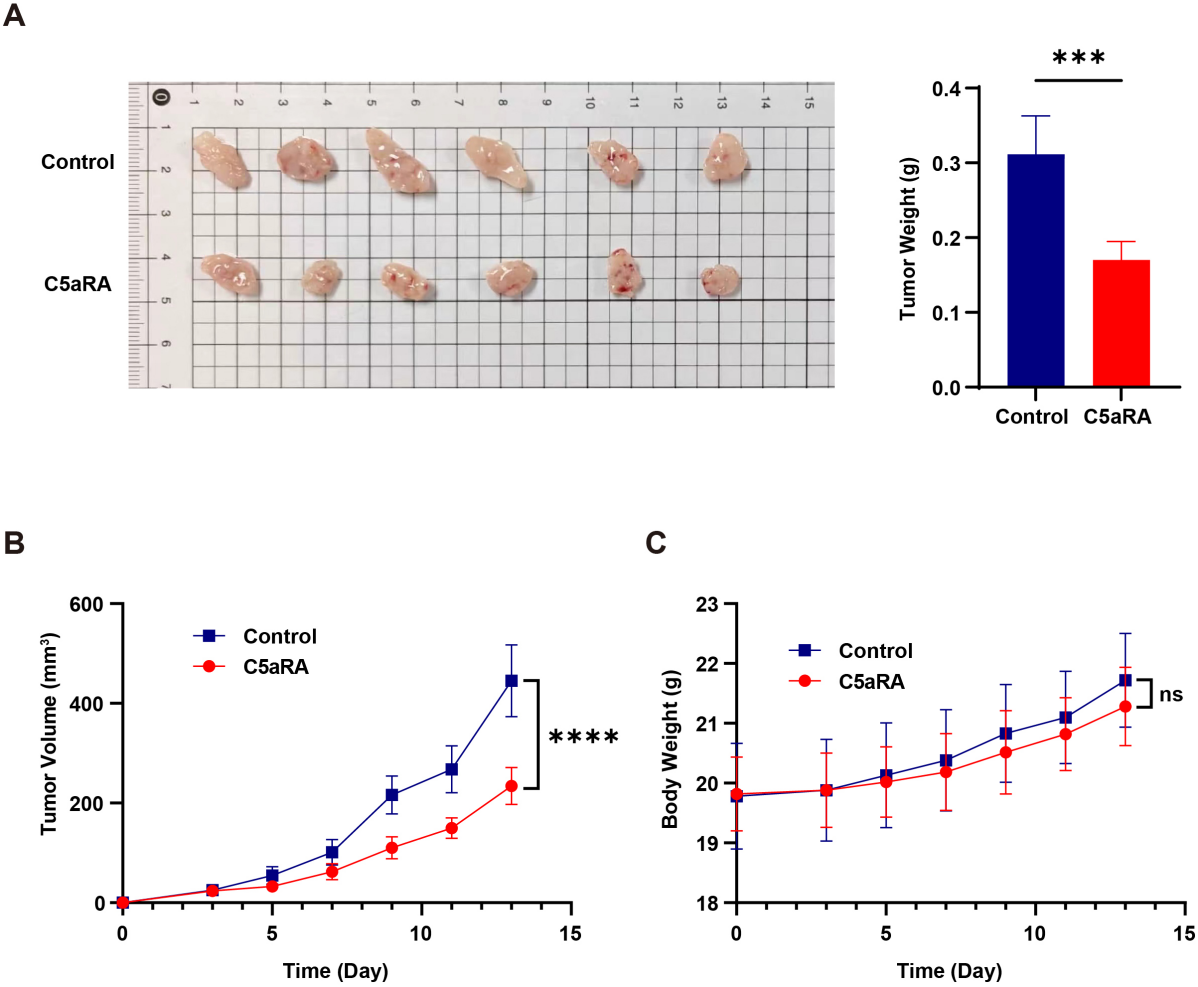
C5aRA inhibited gastric cancer progression in BGC-823 xenograft mouse model. BALB/C female nude mice were 6 weeks old at the experiment’s onset. They were randomly assigned to two groups (n=6) and received subcutaneous transplantation of BGC-823 cells (~2×10^6^ BGC-823 cells into the subcutaneous on back). After tumor formation within three days, for C5aRA group, mice were treated with the C5aR antagonist (C5aRA) (1 mg/kg, intravenously via the tail), every two days, with tumor volume being monitored. While for control group, mice were treated with 0.9% NaCl. **(A, B)** Tumor size, weight and volume of BGC-823 xenograft mice treated with or without C5aRA. **(C)** Body weight of BALB/C nude mice treated with or without C5aRA. Statistical analyses were executed with unpaired two-tailed Student’s t-test. Data are presented as mean ± standard deviation (SD). Significance levels were set at p<0.001 (denoted by ***), and p<0.0001 (denoted by ****).

To further validate the results, we conducted animal experiments in which 2 × 10^6 BGC-823 cells were inoculated into BALB/c nude mice, establishing a nude mouse gastric cancer transplant tumor model. Tumors began to form after three days, and C5aRA was administered via tail vein injection every two days. We monitored the growth of the mice and the transplanted tumors throughout the study. After 15 days, the mice were euthanized, and tumor tissues were excised to assess tumor size and weight. As illustrated in **Figures 6A–C**, the tumor volume and weight of BGC-823 xenografts were significantly reduced following intravenous (i.v.) injection of C5aRA, compared to the control group receiving a 0.9% NaCl solution.

### 3.7 Blocking C5a-C5aR pathway inhibited macrophages polarization to M2 phenotype and downregulated the expression of LCN2 and ER stress

**Figure 7:**
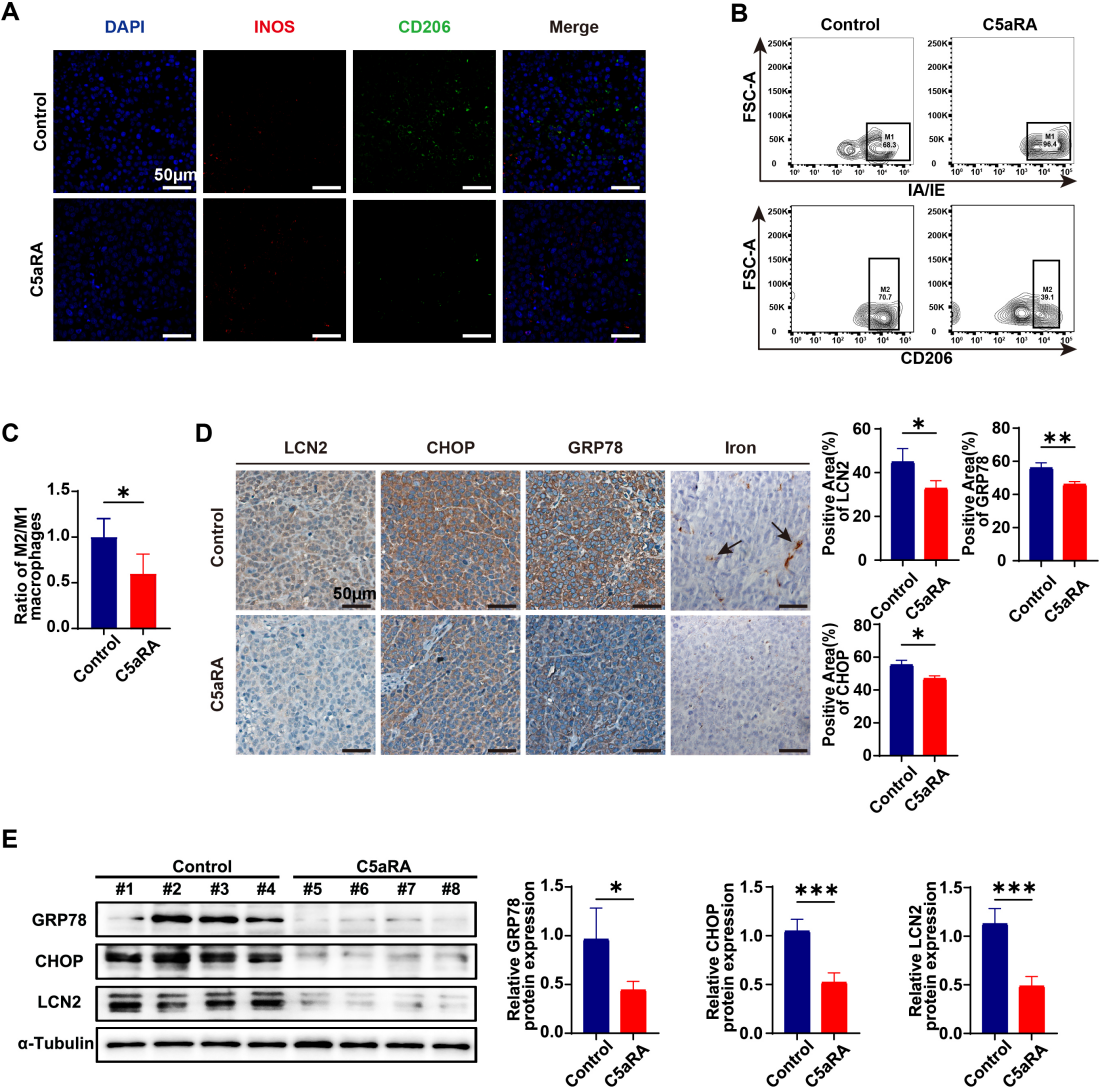
C5aRA prevents the polarization of macrophages towards the M2 phenotype and reduces the expression of LCN2 and ER stress markers. **(A)** Proportion of macrophages in the tumor of two group of xenograft mice (Control and C5aRA) was shown by IF staining (representative panels scale bar, 50 μm). **(B)** The relative proportion and of macrophages in two group of xenograft tumors (Control and C5aRA) was shown by FCM (n=4). **(C)** Statistical analyses of relative proportion of macrophages in xenograft tumors according to FCM results. **(D)** LCN2, CHOP and GRP78 expression in two group of xenograft tumors (Control and C5aRA) were examined using IHC, and statistically analyzed the percentage of positive areas (n=3). Iron deposition in xenograft tumors were examined using DAB enhanced Perls blue staining (representative panels scale bar, 50 μm). **(E)** Protein levels of CHOP, GRP78 and LCN2 in the tumor of two group of xenograft mice (Control and C5aRA) were examined using western blot analysis, results were normalized to the internal control α-tubulin, and the relative protein expression levels were statistically analyzed (n=4). Statistical analyses were executed using GraphPad Prism 9 software, with comparisons made via unpaired two-tailed Student’s t-test. Data are presented as mean ± standard deviation (SD). Significance levels were set at p<0.05 (denoted by *), p<0.01 (denoted by **), p<0.001 (denoted by ***).

To further investigate the role of the C5a-C5aR pathway in gastric cancer iron metabolism, we collected endpoint xenograft specimens from mice to analyze tumor-infiltrating macrophages using immunofluorescence staining and flow cytometry. As illustrated in **Figure 7A**, treatment with C5aRA led to a decrease in the expression of the M2 macrophage marker *CD206* and an increase in the M1 macrophage marker *iNOS*. Additionally, flow cytometry analysis indicated that C5aRA treatment reduced the ratio of M2/M1 macrophages within the tumor microenvironment (**Figures 7B, C**). These findings demonstrated that C5aRA could inhibit gastric cancer development by promoting macrophage polarization toward the M1 phenotype. Immunohistochemistry and Western blot analyses revealed that after C5aRA treatment, the expression of ER stress markers and LCN2 also decreased (**Figures 7D, E**). DAB enhanced Perl’s blue staining confirmed reduced iron deposition in tumor tissue of C5aRA group mice (**Figure 7D**). These results are consistent with our *in vitro* experiments, suggesting that the C5aR antagonist can inhibit M2 polarization and reduce the expression of ER stress markers and LCN2 *in vivo*.

In summary, we propose that the C5a-C5aR pathway facilitates gastric cancer progression by modulating iron metabolism. It promotes the polarization of macrophages toward the iron-releasing M2 phenotype while simultaneously enhancing the expression of the iron-transporter protein LCN2 through ER stress activation. Consequently, gastric cancer cells can obtain sufficient iron from macrophages in the tumor microenvironment to support their proliferative capacity.

## 4 Discussion

Over the past decade, chemotherapy has remained the standard treatment for most patients with unresectable or metastatic gastric cancer. Currently, the primary chemotherapy agents used in clinical practice include fluorouracil, oxaliplatin, capecitabine, and paclitaxel. These drugs can result in a range of adverse reactions, encompassing acute effects like nausea, vomiting, and diarrhea, as well as chronic complications such as bone marrow suppression, hair loss, skin damage, and neurological issues, which ultimately restrict the efficacy of chemotherapy (45). The existing targeted therapies, such as trastuzumab, are monoclonal antibodies that specifically target HER-2, inducing antibody-dependent cytotoxicity, inhibiting HER-2 mediated signaling, and preventing the cleavage of HER-2 extracellular domains (46). While these treatments provide survival benefits for a limited number of HER-2+ tumor patients, the median overall survival (OS) for gastric cancer patients remains low (45). Therefore, new therapeutic strategies must be explored. To identify additional treatment targets, more comprehensive research into the mechanisms underlying the occurrence and progression of gastric cancer is essential. This study focuses on the role of iron metabolism in gastric cancer and investigates potential mechanisms of gastric cancer development through the lens of complement immunology, aiming to inform future treatment approaches for this disease.

As a vital nutrient, iron plays a crucial role in tumor initiation, proliferation, and metastasis (47). Consistent with this notion, a higher risk of cancer has been positively associated with increased dietary iron intake, augmented body iron stores, and genetic conditions of iron overload, such as those found in individuals with hereditary hemochromatosis (19, 47, 48). Molecular studies indicate that elevated iron levels can promote tumor growth, while the use of iron chelators or the blockade of iron import effectively suppress tumor proliferation (19, 49). Notably, distinct iron regulatory mechanisms have been identified in malignant cells; for instance, breast cancer cells can express hepcidin to downregulate ferroportin, thereby reducing cellular iron efflux (12). Furthermore, the overexpression of LCN2 in tumors may serve as a mechanism to enhance iron delivery via 24p3R, addressing the iron demands of the tumors (12, 50, 51).

Macrophages play a crucial role in regulating systemic iron metabolism. During the phagocytosis of senescent erythrocytes in the spleen and liver, macrophages release heme, which is subsequently catabolized by heme oxygenase (mainly HO-1) to produce iron. This recycled heme-derived iron constitutes the majority of available iron in the body, being either stored in ferritin (FT) or transported to ferroportin (FPN) (52). Notably, distinct macrophage phenotypes exhibit differential expression of iron-regulated genes, suggesting that macrophage polarization is linked to alterations in iron homeostasis (33, 53). In the early stages of tumorigenesis, pro-inflammatory cytokines stimulate M1-like macrophages, characterized by low levels of FPN and elevated FT levels, to adopt an iron-sequestering phenotype as an antitumor response. In contrast, M2-like macrophages display an iron-releasing phenotype, featuring higher expression of the iron exporter FPN and reduced levels of the storage protein FT, thereby enhancing iron recycling and its export into the extracellular space (54).

TAMs are increasingly recognized as an anti-inflammatory phenotype that facilitates cancer progression by releasing iron. These cells exhibit high expression levels of CD163, a high-affinity scavenger receptor that forms a complex with haptoglobin and hemoglobin for uptake (55, 56). In this context, TAMs also express elevated levels of FPN and LCN2 for transporting iron to cancer cells. Notably, depletion of the established iron exporter FPN does not affect macrophages iron release capacity in breast cancer model (17). These findings imply the presence of an alternative and cancer-related iron transport pathway in the tumor microenvironment that operates independently of FPN. The inability of FPN to contribute to iron export under these conditions may be attributed to local hepcidin expression, which suppresses its levels. Notably, significant amounts of hepcidin are found both within the tumor microenvironment and systemically in cancer patients (11, 57–59).

LCN2, a member of the LCN family of proteins involved in various cellular functions (60), has been extensively studied due to its close association with tumor development (20). Overexpression of LCN2 has been observed in various cancers, indicating its potential as a therapeutic target (23–26). However, the classification of LCN2 as an oncoprotein or a suppressor protein remains contentious. Some studies suggest that LCN2 promotes cancer growth, while others propose its role as a tumor suppressor (61–64). In gastric cancer, Kubben et al. found that elevated levels of MMP-9/lipocalin-2 complexes in gastric cancer tissues correlate with reduced survival (65). Conversely, Zhixin Huang et al. reported that LCN2 inhibits gastric cancer progression through autocrine modulation of the 24p3R/JNK/c-Jun/SPARC axis (66). Additionally, Sadaaki Nishimura et al. demonstrated that LCN2 downregulation significantly enhances the proliferation, invasion, and migration of gastric cancer cells in epithelial-mesenchymal transition (EMT) type gastric cancer (67). However, the role of LCN2 in iron metabolism during GC progression has not been clarified.

Complement is a crucial component of the human immune system, playing a vital role in defending the body against foreign infections. The activation of the complement system occurs via three pathways: the classical pathway, the bypass pathway, and the lectin pathway. In the tumor microenvironment, abnormal complement activation can drive tumor cell growth and angiogenesis (68). Evidence suggests that complement C5a-C5aR facilitates the development of various tumors, including kidney cancer, rectal cancer, and liver cancer (69–71). Our previous research indicated that complement C5a is frequently activated in gastric cancer tissues and promotes the progression of gastric cancer by binding to C5a receptors (C5aR1) expressed on gastric cancer cells (72). Recent studies have highlighted that C5a is a significant inducer of endoplasmic reticulum (ER) stress, which interacts with its receptor C5aR to elicit a robust ER response and contribute to the development of acute lung injury (73).

In our study, Perls’ Prussian blue staining of gastric cancer samples revealed an increase in iron deposition, indicating abnormal iron metabolism during the progression of gastric cancer. Furthermore, by comparing and analyzing the expression levels of ER stress related indicators and LCN2 in the TCGA cohort, we found that these markers were highly expressed in gastric cancer tissues. Immunohistochemistry and WB experiments on clinical samples further confirmed this finding, indicating that ER stress is elevated within gastric cancer tissues and that the expression of LCN2, an iron transporter, is increased during gastric cancer progression. Next, we confirmed the high expression of C5aR1 in gastric cancer tissue and its correlation with patient prognosis. Therefore, we propose that complement C5aR1 is associated with both the development of gastric cancer and iron deposition. Subsequently, to confirm the relationship between complement C5a-C5aR1 and the development of gastric cancer and iron deposition, we stimulated human macrophage THP-1 with or without C5aR antagonist using recombinant C5a protein. The supernatant was extracted as conditioned medium to culture gastric cancer cells, and cell proliferation experiments and iron content measurements were conducted to verify that C5a-C5aR can promote gastric cancer cell growth through macrophages and accelerate iron accumulation, confirming the correlation between complement system and gastric cancer iron metabolism.

To further investigate the impact of C5a-C5aR1 on iron metabolism in gastric cancer, we conducted macrophage polarization experiments. The QRT-PCR results indicated that stimulation with recombinant C5a protein led to a decrease in M1-related markers in both human and mouse macrophages, while M2 macrophage markers increased. Upon the addition of C5aRA, this trend was partially reversed, confirming that C5a-C5aR1 promotes macrophage polarization toward the M2 phenotype. Given that M2 macrophages are known to facilitate tumor iron transport, we conclude that C5a-C5aR1 accelerates iron transport in gastric cancer by promoting M2 polarization of macrophages. Additionally, we examined the expression of ER stress and LCN2. The results from both QRT-PCR and Western Blot experiments demonstrated that C5a enhances the expression of ER stress markers and LCN2, an effect that can be inhibited by the addition of C5aRA. Further experiments revealed that an ER stress antagonist mimics the action of the C5aRA, suggesting that C5a-C5aR1 promotes LCN2 expression through ER stress activation. Given the above results, we have reason to believe that C5a-C5aR promotes iron transport and proliferation in tumor cells through these two pathways. We subsequently validated the above results *in vivo*. BGC823 xenograft mice model was conducted by subcutaneous injection of BGC823 gastric cancer cells into the back of nude mice. The experimental group was injected with C5aRA via the tail vein. The results indicate that C5aRA inhibits tumor growth *in vivo*. Meanwhile, the results of WB, flow cytometry, immunohistochemistry, and Perls blue staining (DAB enhanced) analysis of tumor tissue showed that C5aRA can inhibit M2 polarization, ER stress, and LCN2 expression, thereby reducing iron deposition in the tumor.

Although abnormal iron metabolism in the tumor microenvironment has been well documented, the influence of the complement system on iron metabolism and its role in the onset and progression of gastric cancer has been underexplored. For the first time, we have linked and validated the complement C5a-C5aR pathway in the immune system with iron metabolism in the tumor environment, providing new insights for future research on tumor iron metabolism. Additionally, our experiments have demonstrated that the C5aRA exerts a significant inhibitory effect on tumor growth in nude mouse models of gastric cancer. This targeting of the complement system may offer innovative approaches for gastric cancer treatment. Currently, numerous drugs aimed at disrupting the interaction between C5a and its receptor are in various stages of development. Among these, the C5a antibody vilobelimab and the small molecule drug avocapan have received FDA approval for the treatment of COVID-19 and antineutrophil cytoplasmic antibody (ANCA)-associated vasculitis. Additionally, Phase I clinical trials have demonstrated that PMX53, a cyclic peptide acting as a C5aR1 antagonist, is safe for oral administration in the treatment of rheumatoid arthritis (76, 77). However, due to the complexity and heterogeneity of tumors, these drugs have not yet been approved for cancer therapy. Our study may provide valuable support for the potential use of these drugs in the clinical treatment of cancer.

However, this study has certain limitations. We have demonstrated that C5a-C5aR pathway could promote macrophage polarization to M2 phenotype, and up-regulate the expression of LCN2 to enhance iron transport from macrophages to gastric cancer cells, thereby accelerating gastric cancer progression. It is worth considering whether this mechanism is applicable to other tumors like kidney cancer, rectal cancer, breast cancer and liver cancer, for C5a-C5aR pathway facilitating the development of these tumors (69–71, 78). However, in head and neck cancer, inhibition of C5a signaling using receptor antagonists accelerated tumor growth (79). Thus, the role of C5a-C5aR pathway in head and neck cancer iron metabolism is worth further research.

Besides, in our study, we conducted the *in vivo* experiments in immunodeficient mice. Immunodeficient mice, lacking a complete immune system, cannot accurately replicate the immune response involved in the development of human gastric cancer. Although the complement system is a crucial component of innate immunity, it also affects adaptive immunity, both of which play significant roles in cancer-related iron metabolism and progression. To better simulate the natural microenvironment of human gastric cancer, it is essential to develop a gastric cancer model using immunocompetent mice for further investigation. Besides, Murine models are instrumental in understanding the mechanisms underlying gastric cancer progression and in exploring new therapeutic strategies. Nonetheless, biological differences, tumor heterogeneity, microenvironment disparities, limited genetic diversity, and challenges in replicating human immune responses present significant barriers to the clinical application of experimental results. Therefore, complementary approaches—such as the use of humanized models, organoids, and clinical data—could help bridge the gap between murine studies and human gastric cancer outcomes.

Targeting cancer iron metabolism is a promising field in tumor therapy, and until now, there are two diametrically opposed strategies focused on iron metabolism. One strategy is reducing iron supply to cancer cells, so inhibiting cancer cell proliferation. Like in our study, blockade C5a-C5aR pathway could decrease iron transport from macrophages to cancer cells. On the contrary, the other strategies are aimed at increasing iron in cancer cells, thus leading to cancer cell ferroptosis, inhibiting cancer progression. However, cancer cells develop many strategies to resist ferroptosis. In gastric cancer cells, Wnt/beta-catenin signaling confers ferroptosis resistance by targeting GPX4 (80). Whether C5a-C5aR pathway could affect cancer cell ferroptosis is also worthy of study. Given the pivotal role of iron metabolism in cancer progression, more research into the underlying mechanisms is imperative for advancing cancer therapy.

**Figure 8:**
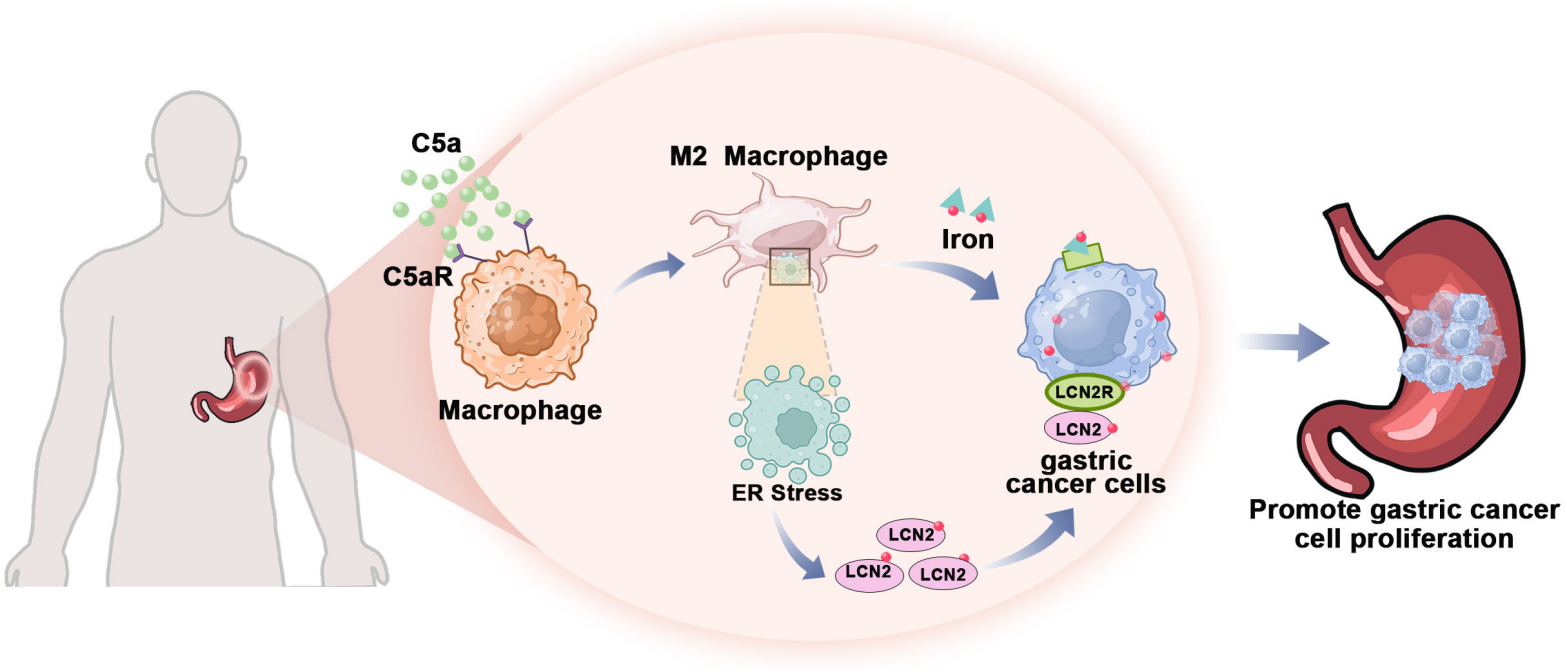
The mechanism by which C5a-C5aR pathway accelerated the progression of gastric cancer through enhancing iron transfer from macrophages to cancer cells. The C5a-C5aR pathway enhances macrophage polarization towards the M2 phenotype while simultaneously increasing the expression of the iron-transporter protein LCN2 through ER stress activation. Consequently, gastric cancer cells acquire sufficient iron from M2 macrophages within the tumor microenvironment to sustain their proliferative capacity.

**Figure 9:**
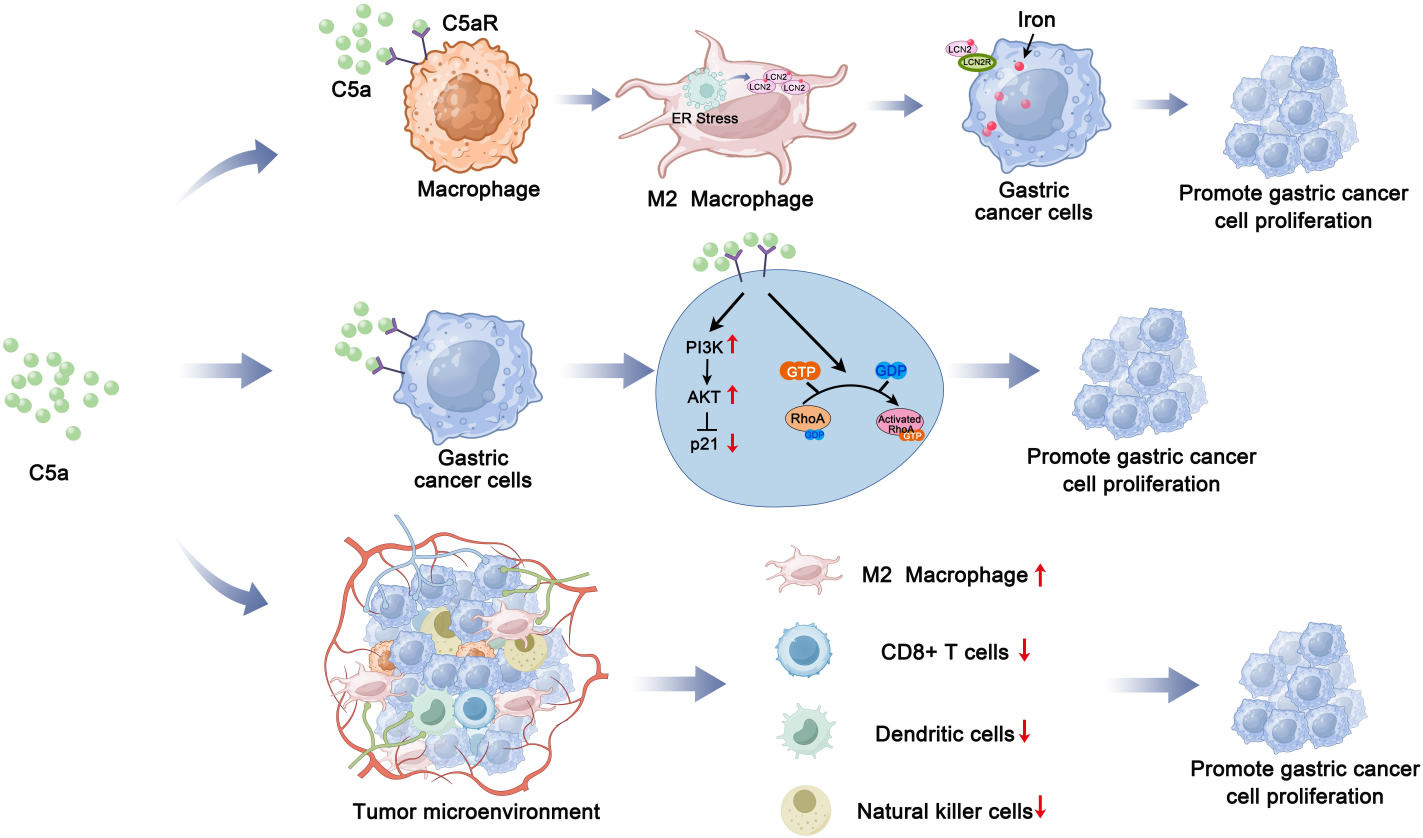
Schematic summarizing the proposed mechanism of the C5a-C5aR pathway’s role in gastric cancer progression.

In summary, our study demonstrated that C5a-C5aR pathway promoted LCN2 up-regulation by activation of ER stress and lead macrophages polarization to M2 phenotype. This process enhances the transport of iron from macrophages to gastric cancer cells, thereby accelerating the progression of gastric cancer (**Figure 8**). Besides influencing iron metabolism, our previous study also proved that C5a-C5aR pathway potentiated the pathogenesis of gastric cancer by down-regulating p21 expression (72). And in Takayoshi Kaida study, C5a receptor (CD88) promoted motility and invasiveness of gastric cancer by activating RhoA (74). Honghong Shen also demonstrated that C5aR1 shaped a non-inflammatory tumor microenvironment and mediates immune evasion in gastric cancer (75). According to these studies, we construct a schematic diagram summarizing the proposed mechanism of the C5a-C5aR pathway’s role in gastric cancer (**Figure 9**).

## Data Availability

The raw data supporting the conclusions of this article will be made available by the authors, without undue reservation.
